# Right ventricular function parameters in pulmonary hypertension: echocardiography vs. cardiac magnetic resonance

**DOI:** 10.1186/s12872-020-01548-4

**Published:** 2020-06-01

**Authors:** Anna Werther Evaldsson, Anthony Lindholm, Raluca Jumatate, Annika Ingvarsson, Gustav Jan Smith, Johan Waktare, Göran Rådegran, Anders Roijer, Carl Meurling, Ellen Ostenfeld

**Affiliations:** 1 Lund University, Department of Clinical Sciences Lund, Cardiology, Section for Heart Failure and Valvular Disease, Skane University Hospital, Lund, Sweden; 2 Lund University, Department of Clinical Sciences Lund, Clinical Physiology, Skane University Hospital, Lund, Sweden; 3 Liverpool Heart and Chest Hospital, Liverpool, UK

**Keywords:** Echocardiography, Cardiac magnetic resonance, Right ventricular function, Validation study

## Abstract

**Background:**

Right ventricular (RV) function is a major determinant of outcome in patients with pulmonary hypertension. Cardiac magnetic resonance (CMR) is gold standard to assess RV ejection fraction (RVEF_CMR_), however this is a crude measure. New CMR measures of RV function beyond RVEF_CMR_ have emerged, such as RV lateral atrio-ventricular plane displacement (AVPD_lat_), maximum emptying velocity (S’_CMR_), RV fractional area change (FAC_CMR_) and feature tracking of the RV free wall (FWS_CMR_). However, it is not fully elucidated if these CMR measures are in parity with the equivalent echocardiography-derived measurements: tricuspid annular plane systolic excursion (TAPSE), S’-wave velocity (S’_echo_), RV fractional area change (FAC_echo_) and RV free wall strain (FWS_echo_). The aim of this study was to compare regional RV function parameters derived from CMR to their echocardiographic equivalents in patients with pulmonary hypertension and to RVEF_CMR_.

**Methods:**

Fifty-five patients (37 women, 62 ± 15 years) evaluated for pulmonary hypertension underwent CMR and echocardiography. AVPD_lat_, S’_CMR_, FAC_CMR_ and FWS_CMR_ from cine 4-chamber views were compared to corresponding echocardiographic measures and to RVEF_CMR_ delineated in cine short-axis stack.

**Results:**

A strong correlation was demonstrated for FAC whereas the remaining measurements showed moderate correlation. The absolute bias for S’ was 2.4 ± 3.0 cm/s (relative bias 24.1 ± 28.3%), TAPSE/AVPD_lat_ 5.5 ± 4.6 mm (33.2 ± 25.2%), FWS 4.4 ± 5.8% (20.2 ± 37.5%) and for FAC 5.1 ± 8.4% (18.5 ± 32.5%). In correlation to RVEF_CMR,_ FAC_CMR_ and FWS_echo_ correlated strongly, FAC_echo_, AVPD_lat_, FWS_CMR_ and TAPSE moderately, whereas S’ had only a weak correlation.

**Conclusion:**

This study has demonstrated a moderate to strong correlation of regional CMR measurements to corresponding echocardiographic measures. However, biases and to some extent wide limits of agreement, exist between the modalities. Consequently, the equivalent measures are not interchangeable at least in patients with pulmonary hypertension. The echocardiographic parameter that showed best correlation with RVEF_CMR_ was FWS_echo_. At present, FAC_echo_ and FWS_echo_ as well as RVEF_CMR_ are the preferred methods to assess and follow up RV function in patients with pulmonary hypertension. Future investigations of the CMR right ventricular measures, beyond RVEF, are warranted.

## Background

Right ventricular (RV) function is a major determinant of outcome in patients with pulmonary hypertension [1, 2]. Comprehensive assessment of RV function is challenging and measurements beyond volumetric changes, such as RV ejection fraction (EF), are eligible [1, 3, 4]. However, RVEF is a crude measure and more subtle methods are warranted for assessing patients with pulmonary hypertension.

First-line modality for evaluation of cardiac morphology and function in clinical practice is echocardiography. Nevertheless, RV assessment by echocardiography is vulnerable to the complex geometry of the RV [5, 6]. The drawback of most of the echocardiographic measurements is that they are estimates of longitudinal function. Since approximately 80% of the RV stroke volume is generated from longitudinal contribution [4], in clinical use they are considered to reflect global RV function. However, in patients with a pressure overloaded RV, the contraction pattern changes significantly from primarily longitudinal to circumferential contraction [7]. Conventionally, RV function by echocardiography is estimated by tricuspid annular plane systolic excursion (TAPSE), S’-wave velocity (S’_echo_), RV fractional area change (FAC_echo_) and myocardial strain derived from the RV lateral free wall (FWS_echo_) [3]. FWS_echo_ has shown promising results in assessment of RV longitudinal systolic function [8–11] and has consequently been included in guidelines [3]. In clinical practice, FAC_echo_ is more commonly used as a surrogate to RVEF [3].

Cardiac magnetic resonance imaging (CMR) is considered “gold standard” for assessing RV volumes as well as ejection fraction (RVEF_CMR_) [12, 13] and is advantageous as it overcomes the echocardiographic limitations [5, 14]. New CMR measures of RV function equivalent to echocardiography-derived measurements have emerged beyond RVEF_CMR_ such as; RV atrio-ventricular plane displacement in the lateral wall (AVPD_lat_) [4, 15], maximum emptying velocity derived from the AVPD curve (S’_CMR_) [16], RV fractional area change (FAC_CMR_) [17] and RV free wall strain (FWS_CMR_) [18]. FWS_CMR_ has been shown to have an added value to RVEF_CMR_ in patients with pulmonary hypertension [19]. However, it is not fully explored how these measures are in parity with the echocardiographic equivalents and to what extend they relate to RVEF_CMR_. The aim of this study was to compare conventional and new RV function parameters derived from CMR to their echocardiographic equivalents in patients with pulmonary hypertension. Secondly, we aimed to investigate how these measures are associated with RVEF_CMR_.

## Methods

### Patient population

Between January 2012 and July 2017 at Skåne University Hospital, Lund, 90 consecutive patients with pulmonary hypertension were evaluated prospectively with echocardiography and CMR [11]. Diagnosis of pulmonary hypertension were set by right heart catheterization and was defined as mean pulmonary arterial pressure ≥ 25 mmHg and was characterized as precapillary when pulmonary artery wedge pressure < 15 mmHg according to current guidelines [20]. Medical records were used for retrieving patient characteristics. Patients were included if ≤14 days between CMR and echocardiography given no change in medical treatment or clinical status during this time. Patients were excluded if atrial fibrillation was present (*n* = 15) or if echocardiographic image quality were inadequate (*n* = 20) owing to full visualization of the RV was not possible despite meticulous attempts (*n* = 8) or when the endocardial borders were not traceable for FAC_echo_ and FWS_echo_ measurements (*n* = 12).

### Echocardiography

**Fig. 1:**
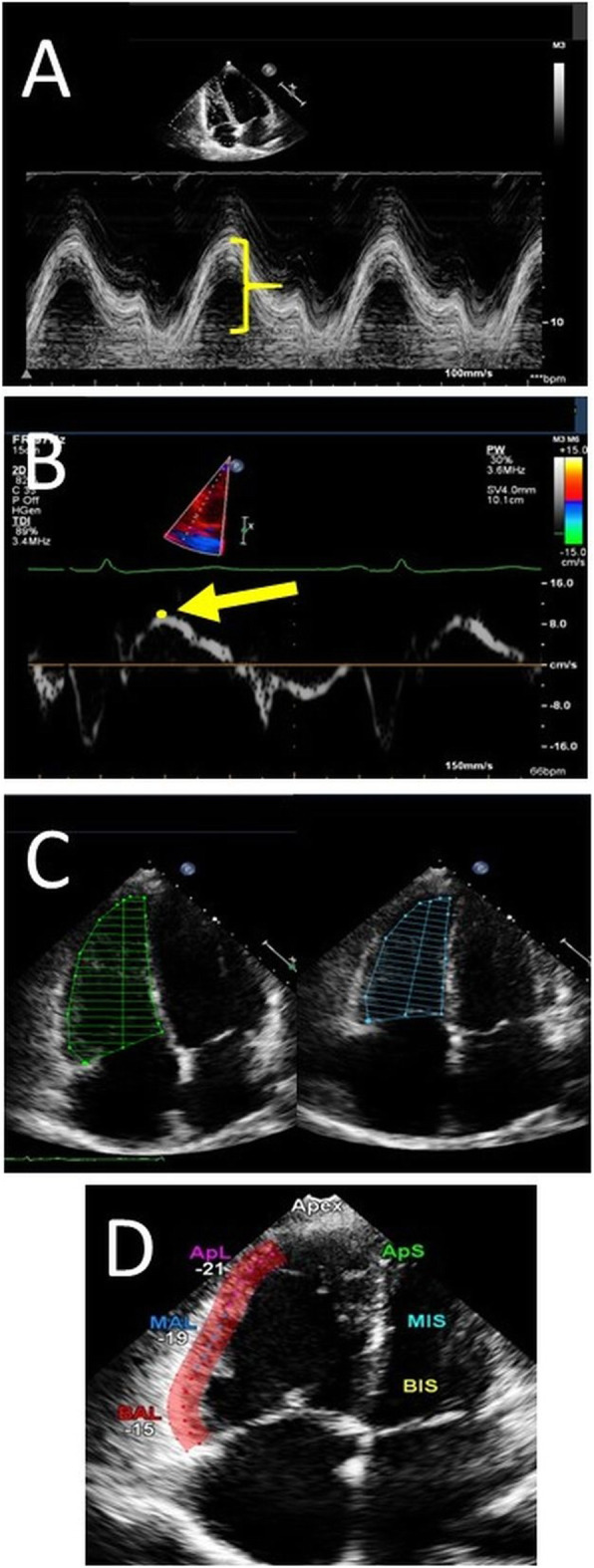
Illustration of echocardiographic measures for assessing right ventricular function. **a**: TAPSE (tricuspid annular plane systolic excursion), **b**: S’_echo_ (S’-wave velocity), **c**: FAC_echo_ (fractional area change), **d**: FWS_echo_ (free wall strain)

Echocardiographic images were obtained by an S5–1 transducer using an iE33 platform (Philips Healthcare, Eindhoven, NL). Acquisition and assessment were performed according to guidelines [3], by senior sonographers. RV function was assessed by the conventional parameters TAPSE, S’_echo_, FAC_echo_ and FWS_echo_ according to current guidelines from an apical RV focused 4-chamber view using Xcelera (Philips Healthcare, Eindhoven, NL) (Fig. 1) [3]. Framerate for 2-D images was 50–80 Hz. A dedicated software for strain analysis was used for offline analysis (CMQ, Q-lab 10.3, Philips Healthcare). At end-diastole the region of interest was traced along the RV endocardium and a propagated tracking was performed. Manual correction was performed if there was inappropriate tracking. Calculation of FWS_echo_ was made as an average of the three peak systolic strains values along the RV free wall. Low intra- and inter-observer variability within our research group has previously been validated and published [10].

### Cardiac magnetic resonance imaging

**Fig. 2:**
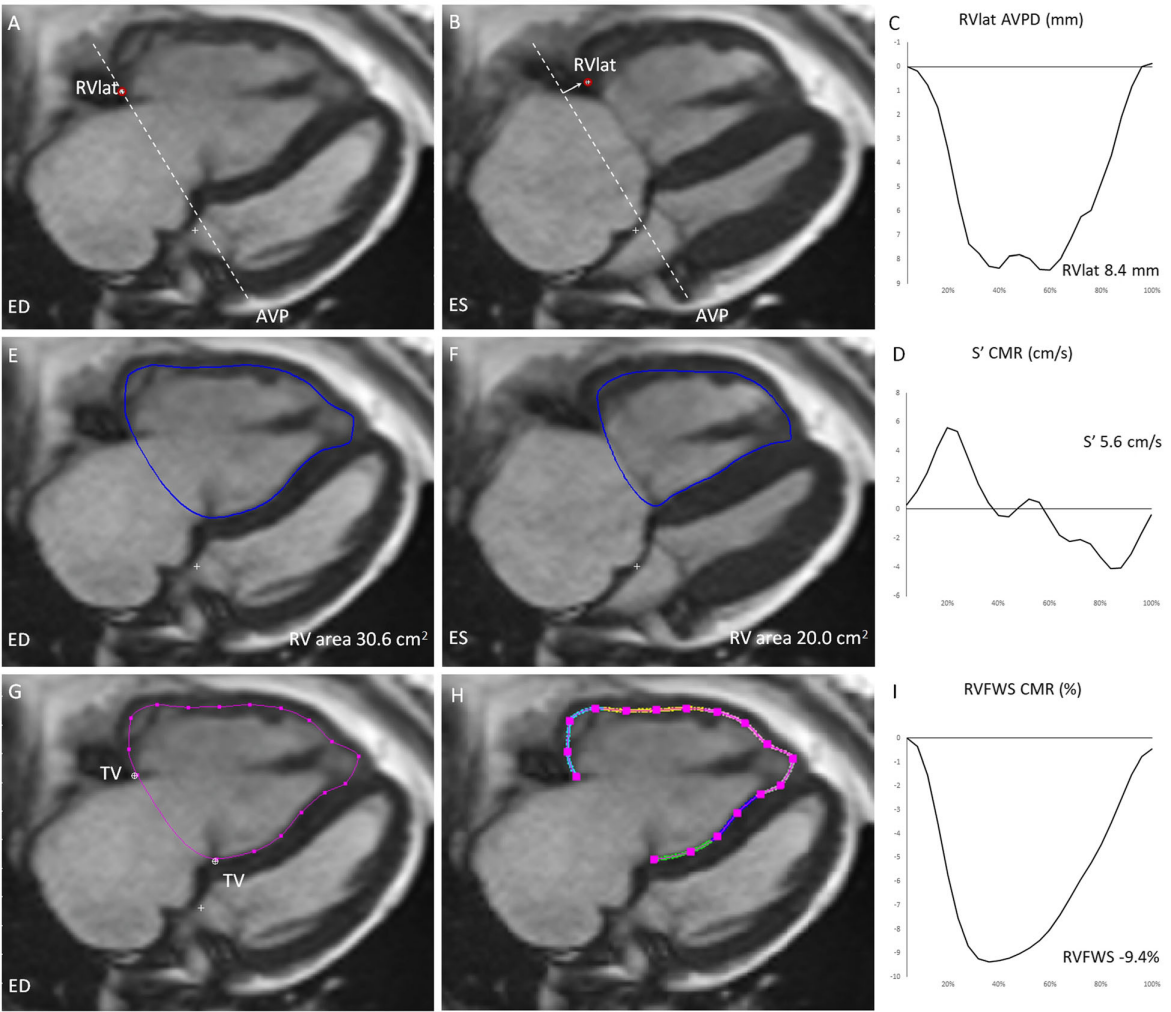
Illustration of novel CMR derived measures for assessing right ventricular (RV) function in a patient with pulmonary hypertension. **a**: RV_lat_ (RV lateral free wall, red full circle) in end-diastole (ED) at the AVP (atrio-ventricular plane, dashed line), **b**: RV_lat_AVP displacement (AVPD_lat_) in end-systole (ES) is marked with a white arrow, **c**: Time-resolution AVPD curve over one cardiac cycle, **d**: S’_CMR_ (max emptying velocity) derived from the AVPD curve, FAC_CMR_ (fractional area change) derived from RV area in ED (**e**) and ES (**f**), FWS_CMR_ (free wall strain) derived from tricuspid valve points (TV) and RV endocardial delineation (**g**) in a feature tracking algorithm (**h**) generating a longitudinal strain curve (**i**)

CMR was obtained as described previously by our research group [11]. CMR images were assessed using Segment version 2.2 software (http://segment.heiberg.se) [21]. The volumes of the right ventricle and RVEF were derived from manual delineations of end-diastolic and end-systolic endocardial borders in the short-axis stack. RV measurements were traced based on endocardial delineations. Trabeculation and papillary muscles were included in the RV-volumes according to standardized interpretation [22]. The difference between the end-diastolic and end-systolic volumes divided by the end-diastolic volume were used for RVEF_CMR_ calculation. AVPD_lat_, S’_CMR_, FAC_CMR_, and FWS_CMR_, were analyzed in the 4-chamber view. AVPD_lat_ was quantified from a manual input point at the base of the RV free wall in end-diastole with time-resolved automated tracking through the cardiac cycle (Fig. 2a), and S’_CMR_ was computed as the maximum emptying velocity derived from the time-resolved AVPD curve [16, 23] (Fig. 2b). FAC_CMR_ (Fig. 2c) and FWS_CMR_ was assessed by tracing the RV endocardium at end-diastole. FWS_CMR_ was computed from automated propagated tracking from end-diastole throughout the heart cycle, averaging three regional segmental peak systolic strain values along the RV free wall with manual correction if necessary (Fig. 2d). Low intra- and inter-observer variability within our research group has previously been validated and published [13, 16, 23].

### Statistical analysis

Continuous data was expressed as mean ± standard deviation (SD) or median with inter-quartile range [IQR], as appropriate according to normal distribution. Normality was assessed visually from histograms. Categorical data was expressed in absolute numbers and proportion (percentage). Pearson’s correlation coefficient (r-values) was used for association between echocardiography and CMR. Degree of correlation between tests was classified as either weak (r = 0.3–0.5), moderate (0.5–0.7), strong (0.7–0.9) or very strong (0.9–1.0) [24]. Bias according to Bland-Altman was computed between modalities [25]. A univariate T-test analysis was performed to detect differences between sexes and between patients with and without comorbidities, and a multivariate regression analysis weas performed adjusting of age, sex and co-morbidities. T-test and Fischer’s exact test were used for sensitivity analysis between the patients with PAH and the patients without PAH. Values of two-tailed *P* < 0.05 were considered statistically significant. Analyses were performed using a commercially available software (IBM, SPSS Statistics, version 25, Chicago, IL, USA).

## Results

### Clinical characteristics

Baseline characteristics of the included 55 patients (37 women, 62 ± 15 years) are shown in Table 1. The median time between echocardiography and CMR was 1 day [1–2 days].

**Table 1:** Baseline characteristics for demographic, clinical and laboratory parameters

Number of patients	55
Sex (women/men)	37/18 (67/33)
Age (years)	62 ± 15
BSA (m^2^)	1.9 ± 0.2
Etiology of pulmonary hypertension	
Group I (pulmonary arterial hypertension, PAH)	40 (73)
Group II (due to left heart disease)	5 (9)
Group III (due to lung diseases and/or hypoxia)	1 (2)
Group IV (due to chronic thromboembolism)	9 (16)
Group V (unclear and/or multifactorial mechanisms)	0 (0)
Laboratory parameters	
NT-proBNP (ng/L)	979 [329–2286]
Saturation peripheral (%)	93 ± 4
Comorbidites	
Diabetes	14 (26)
Hypertension	13 (24)
Coronary artery disease	7 (13)
Chronic obstructive pulmonary diseases	7 (13)
Obstructive sleep apnea syndrome	1 (3)
6-min walking test (m)	325 [200–412]
Functional class, NYHA	
I	3 (6)
II	29 (53)
III	19 (35)
IV	4 (7)
Medication	
O_2_	14 (26)
Diuretics	26 (47)
Calcium antagonists	10 (18)
ACE/ARB blockers	13 (24)
Beta blockers	14 (26)
Nitrates	3 (6)
ERA	8 (15)
PDE5I	8 (15)
Prostanoids	1 (2)
sGCS	1 (2)

Data are expressed as mean ± SD, median [inter-quartile range] or as number (percentage). *BSA* Body surface area, *NT-proBNP* brain natriuretic peptide, *ACE* Angiotensin-Converting Enzyme, *ARB* Angiotensin Receptor Blockers, *ERA* Endothelin Receptor Antagonist, *PDE5I* Phosphodiesterase type 5 inhibitors, *sGCS* Guanylate Cyclase Stimulators

Of the five etiological subclasses of pulmonary hypertension [20], the most common etiologies were pulmonary arterial hypertension (*n* = 40) and chronic thromboembolic pulmonary hypertension (*n* = 9). Median NT-proBNP was 979 ng/L [329–2286 ng/L]. 18% of the patients were treated with pulmonary arterial vasodilators (60% of these with double or triple medication). 87% of the patients were in WHO-functional class II or III and the median 6-min walk test was 325 m [200–412]. Comorbidities were present in 28 patients (Table 1). Echocardiographic characteristics are shown in Table 2. Echocardiographic parameters were normal concerning TAPSE and S’_echo_ but reduced for FAC_echo_ and FWS_echo_. Regarding CMR parameters, RVEF_CMR_ was reduced compared to reference values, whilst AVPD_lat_ was normal [26, 27]. Echocardiographic and CMR parameters measuring RV systolic function are shown in Table 3. Regarding factors such as sex and comorbidities a slightly lower LVEDV and LVESV were present in women compared to men, and a lower FWS_CMR_ could be shown in patients with compared with those without comorbidities. When adjusting for age, sex and comorbidities using a multilinear regression analysis, FWS_CMR_ was the only parameter that was affected (*p* = 0.019). No differences were seen between the patients with PAH (*n* = 40) and those without PAH (*n* = 15) (Supplemental table 1).

**Table 2:** Echocardiographic characteristics

**Left heart**	
IVSd (mm)	9.8 ± 2.4
LVIDd (mm)	42 ± 10
LVPWd (mm)	8.7 ± 2.0
LVEDV (mL)	77 ± 36
LVESV (ml)	32 ± 21
LVEF (%)	60 ± 13
LA Volume/BSA (mL/m^2^)	28 ± 14
**Right heart**	
RA volume/BSA (mL/m^2^)	39 ± 23
RA area (cm^2^)	22 ± 7
RVDd	37 ± 6
RV size inflow (mm)	48 ± 9
RV size mid cavity (mm)	38 ± 10
TR (0–3)	1.0 [0.5–1.5]
TR gradient (mmHg)	56 ± 21
IVCd (mm)	19 ± 6

Data are expressed as mean ± SD or median [inter-quartile range]. *IVSd* Intra ventricular septum diameter, *LVIDd* Left ventricular inner diastolic diameter, *LVPWd* Left ventricular posterior wall diameter, *LVEDV* Left ventricular end-diastolic volume, *LVEF* Left ventricular ejection fraction, *LVESV* Left ventricular end-systolic volume, *LA* Left atrium, *RA* Right atrium, *RVDd* Right ventricular diastolic diameter, *RVEDA* Right ventricular end diastolic area, *RVESA* Right ventricular end systolic area, *RV* Right ventricle, *TR* Tricuspid valve regurgitation, *TR gradient* Trans-tricuspid gradient, *IVCd* Inferior vena cava diameter

**Table 3:** Correlation of echocardiographic and cardiac magnetic resonance imaging measures of right ventricular function

	Mean ± SD (n)	Correlation with corresponding CMR parameter, r-value	Correlation with RVEF_CMR_, r-value
CMR			
RVEF (%)	42 ± 14 (55)		
AVPD_lat_ (mm)	14 ± 4 (54)		0.736^***^
S’_CMR_ (cm/s)	8.8 ± 3.0 (55)		0.472^***^
FAC_CMR_ (%)	35 ± 13 (55)		0.819^***^
FWS_CMR_ (%)	−20.1 ± 7.7 (55)		0.726^***^
Echocardiography			
TAPSE (mm)	19 ± 6 (53)	0.644^***^	0.592^***^
S’_echo_ (cm/s)	11.3 ± 3.5 (53)	0.599^***^	0.385^**^
FAC_echo_ (%)	30 ± 14 (55)	0.808^***^	0.681^***^
FWS_echo_ (%)	−15.7 ± 4.8 (55)	0.656^***^	0.778^***^

Data is presented as mean ± SD. For correlation between the methods, Pearson’s correlation coefficients were used. Significant correlation is present when: **: *p* < .01, ***: *p* < 0.001. *CMR* Cardiac magnetic resonance, *RVEF* Right ventricular ejection fraction derived from CMR, *AVPD*_*lat*_ Lateral atrio-ventricular plane displacement, *S’* S’-wave velocity, *FAC* Fractional area change, *FWS* Right ventricular free wall strain, *TAPSE* Tricuspid annular plane systolic excursion

### Correlation between CMR and echocardiographic measures

**Fig. 3:**
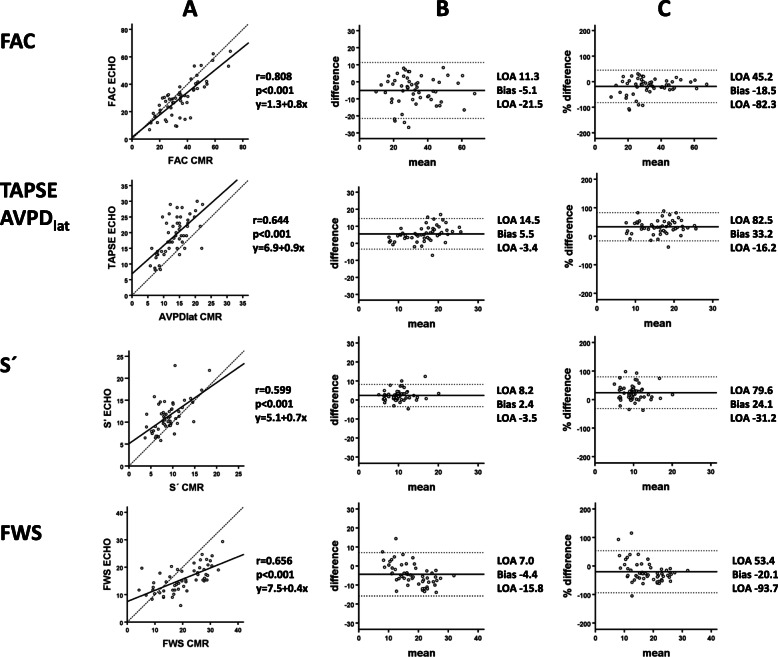
**a**: Scatterplots with regression lines delineates the correlation between right ventricular (RV) function parameters derived by CMR and echocardiography (echo). The dotted line represents the reference line. **b**: Bland-Altman plots for illustrating agreement of RV function parameters as measured by CMR and echocardiography. **c**: Bland-Altman plots based on the percentage differences in RV function parameters measured by CMR and echocardiography. AVPD_lat_ (lateral atrio-ventricular plane displacement), S’_CMR_ (max emptying velocity), FAC (fractional area change), RVFWS (free wall strain), LOA (level of agreement).

**Fig. 4:**
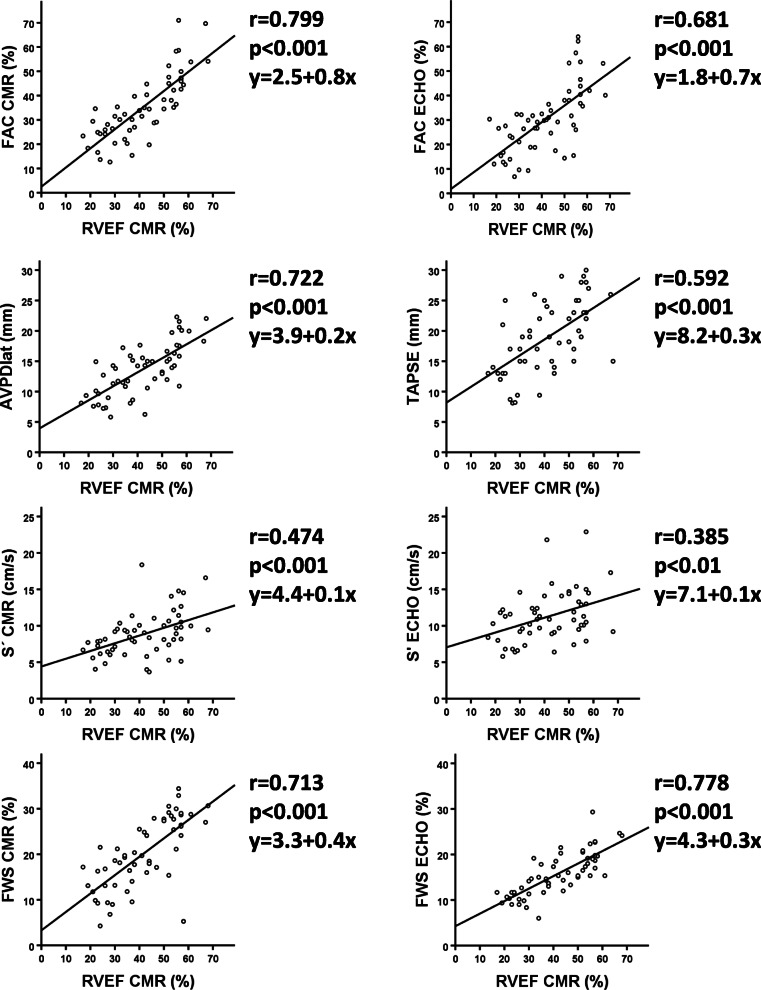
Scatterplots with regression lines for correlation between right ventricular (RV) ejection fraction and function parameters derived from CMR and echocardiography. TAPSE (tricuspid annular plane systolic excursion), S’ (S’-wave velocity), FAC (fractional area change), FWS (free wall strain), AVPD_lat_ (lateral atrio-ventricular plane displacement), S’_CMR_ (max emptying velocity)

R-values were 0.808 for FAC_CMR_ vs. FAC_echo_, 0.656 for FWS_CMR_ vs. FWS_echo_, 0.644 for AVPD_lat_ vs. TAPSE, and 0.599 for S’_CMR_ vs. S’_echo_, (*p* < 0.0001 for all) (Table 2, Fig. 3). In comparison with RVEF_CMR_, the parameters FAC_CMR_ and FWS_echo_ demonstrated a strong correlation, whilst a moderate correlation was demonstrated with FAC_echo_, AVPD_lat_, FWS_CMR_ and TAPSE. A weak correlation with RVEF_CMR_ was demonstrated for S’ measured by both modalities (Table 2, Fig. 4).

CMR measurements differed significantly in absolute values compared to echocardiographic measures, with S’_CMR_ and AVPD_lat_ being lower and FWS_CMR_ and FAC_CMR_ higher (Table 2, Fig. 3). The absolute bias for S’ was 2.4 ± 3.0 cm/s (relative bias 24.1 ± 28.3%), TAPSE/AVPD_lat_ 5.5 ± 4.6 mm (33.2 ± 25.2%), FWS 4.4 ± 5.8% (20.2 ± 37.5%) and for FAC 5.1 ± 8.4% (18.5 ± 32.5%).

## Discussion

To our knowledge, this is the first comprehensive study comparing conventional and new RV function parameters derived from CMR to their echocardiographic equivalents in patients with pulmonary hypertension. Our results indicate strong to moderate correlation of CMR measures of RV function to corresponding echocardiographic parities. However, there are biases that needs to be addressed. As such, lowest relative bias was shown for FAC, then FWS, S’ and highest was for TAPSE/AVPD_lat_. In comparison with RVEF_CMR_, a strong correlation was demonstrated for FAC_CMR_ and FWS_echo_, whilst a moderate correlation was demonstrated for FAC_echo_, AVPD_lat_, FWS_CMR_ and TAPSE, respectively. Only a weak correlation was demonstrated for S’ measured by both modalities.

### Comparison between corresponding echocardiographic and CMR measurements

Our results demonstrated a moderate correlation for AVPD_lat_/TAPSE. These are measures of longitudinal contraction, and the longitudinal contribution accounts for approximately 80% of the RV stroke volume in normal hearts [4]. TAPSE is widely used for echocardiographic assessment of RV function [3], however the value as a prognostic marker in pulmonary hypertension has been debated and has been withdrawn as a risk marker [28]. As the amplitude of TAPSE can be caused by a passive translation movement (i.e. pseudo-normalisation), it can be questioned as a marker of true RV systolic function in pulmonary hypertension [29]. Other reasons to defer using TAPSE as a risk marker in the guidelines is the angle dependency. AVPD_lat_ is a relatively new and sparsely evaluated method, and not referenced in consensus documents for CMR [22]. We found AVPD_lat_ to be decreased in patients with pulmonary hypertension in alignment with a prior study [15, 17], however, the prognostic value of altered AVPD_lat_ in pulmonary hypertension remains to be investigated.

In our study, the highest bias was demonstrated for TAPSE/AVPD_lat_ with lower absolute values by CMR compared to echocardiography. This is an unexpected finding since AVPD_lat_ is considered to be less angle dependent than the measurements of TAPSE. Consequently, the bias is probably methodological since they are measured from differently obtained 4-chamber views. In CMR, acquisition of the 4-chamber view is planned from the short-axis view to perfectly rotated into the RV focused view [3]. These adjustments are not possible with echocardiography. Furthermore, the differences in temporal resolution (i.e. framerate is higher using echocardiography) could explain some of the bias between modalities.

S’ showed a moderate correlation between echocardiography and CMR. S’_CMR_ has only been described twice previously [16, 23] and this is the first study to compare S’_CMR_ to the echocardiographic equivalent. The disadvantage of S’_echo_ not being representative for RV global function is substantial since it only measures the velocity in one specific point in the basal segment of the RV lateral wall [3]. Both S’_echo_ and TAPSE have in previous studies been presented within normality in pulmonary hypertension even when other RV parameters were impaired [10, 11]. Methodologically, S’_CMR_ is derived from the AVPD curve and is defined as the highest measured instantaneous velocity measured as the steepest systolic slope of the AVPD curve. The velocity at peak emptying (S’_CMR_) could hence be considered as in parity to the peak systolic annular velocity from echocardiography S’_echo_ [16]. S’_CMR_ diminishes some of the limitations for S’_echo_ such as angle-dependency and image quality. On the other hand, S’_CMR_ is acquired with a lower framerate than S’_echo_ and with a subsequent post processed smoothing of the curve. In comparison, S’_echo_ is derived from pulsed-wave tissue-doppler imaging and with a high frame rate. The two methods cannot be considered methodologically fully interchangeable. In our study, S’ showed the best agreement with narrow limits in absolute values between the methods. However, as with the other methods there was a systematic substantial relative bias and broad limits of agreement.

The strongest correlation between the modalities were shown by FAC and to our knowledge this is the first study to compare the two modalities. FAC_echo_ reflects both radial and longitudinal contribution to the overall RV function [3] and predicts both morbidity and mortality [30] in patients with pulmonary hypertension. Compared to echocardiographic assessment, delineation of the heavily trabeculated compact endocardial border is less challenging by CMR. One could argue that even if FAC is a surrogate for RVEF and there seems to be little point in measuring FAC by CMR. However, if RVEF is not applicable from CMR images owing to substantial artefacts or missing slices, FAC appears to be a good alternative to RV functional assessment.

In this study, FWS showed a moderate correlation between modalities. Prior studies have shown conflicting evidence as some are in concordance with ours [31], while others have found a stronger correlation [32, 33]. Strain values were on average 20% higher when measured by CMR with a greater spread of observed values (Fig. 3). The bias (absolute and relative) seems to be more pronounced in patients with lower strain values. There are several methodological reasons for strain values to differ between the modalities [34]. Theoretically, CMR strain assessment is advantageous to echocardiography as it is not vulnerable to poor acoustic windows [14]. However, this explanation for the bias in our study is unlikely since patients with echocardiographic poor acoustic window were excluded. One other reason could be that the software’s are using different kinds of strain, i.e. CMR measures endocardial strain and the echocardiographic software uses mid-myocardial strain. The results in our study implies that echocardiographic and CMR evaluation of RV strain cannot be regarded as interchangeable.

### Comparison with RVEF_CMR_

While RVEF is invaluable in assessment of pulmonary hypertension, it is not a direct measure of RV contractility, RVEF requires augmentation with other global and regional parameters to assess the function on a myocardial level [35, 36]. This forms a rationale for investigating the correlation between RVEF_CMR_ and other measurements parameters.

Our results are in concordance with previous studies on FWS_echo_ and FAC_echo/CMR_ [11, 17] presenting a strong correlation with RVEF_CMR_ with a modest correlation for TAPSE/AVPD_lat_ and a weak correlation for S’_echo_ compared to RVEF_CMR_ [11, 17]. As expected, the parameters measuring of area and volume change are closely related. In alignment with our study others have demonstrated a moderate correlation of RVEF_CMR_ to AVPD_lat_ [17] and FWS_CMR_ [31, 37]. The poor correlation between longitudinal measurements of RV function and RVEF_CMR_ could be explained that RVEF is more related to fractional transverse movements than to longitudinal movements in patients with pulmonary hypertension [38]. This could also explain the strong correlation demonstrated between FAC and RVEF_CMR_.

To our knowledge, this is the first study to evaluate the poor relationship of RVEF_CMR_ to S’_CMR_, and to explore a comprehensive paired comparison of equivalent parameters of echocardiography and CMR.

### Clinical implications

RV function is an important determinant of outcome in patients with pulmonary hypertension [1, 2] and RVEF_CMR_ is a robust and precise method for risk assessment and clinical follow up. However, RVEF_CMR_ is an incomplete method of assessing myocardial contraction as it only evaluates relative volume changes. Several studies have shown regional functional assessment of the RV has incremental value beyond EF [11, 15, 19]. Longitudinal function has been proven of relevance for outcome irrespectively and independently of EF concerning the left ventricle [35, 39, 40]. Regarding RV, limited prognostic data is available at present. At present in clinical practice FAC_echo_, TAPSE, S’_echo_ and FWS_echo_ [3], and to some extent AVPD_lat_ are used for assessment of systolic function although their prognostic values are not fully explored. FAC_CMR_, FWS_CMR_ and S’_CMR_ are new measures that are not yet implemented in clinical practice. To some extent the echocardiographic parameters have been showed to be associated to clinical outcome in pulmonary hypertension [3, 41], while the CMR equivalents are yet to be assessed, although data is accumulating [35, 36].

According to clinical practice in our center, the majority of patients evaluated for pulmonary hypertension are investigated within CMR of two main reasons: to exclude presence of congenital heart disease (including intracardial shunts) and also to have accurate measurements for RVEF and RV size since CMR is considered as gold standard for this purpose and these measures carry prognostic information [42, 43]. CMR are included in the current guidelines [20] when assessing the RV and our center is following the guidelines in that context. Our data implies that the utilization of both imaging modalities of regional measures can helpfully augment global assessment of RV function by RVEF_CMR_, but the equivalent measures are not interchangeable at least in pulmonary hypertension. Clinical outcome data was not within the scope of this study. However, investigating which of the different RV functional markers in the present study is most predictive of outcome is of interest for future studies.

### Limitations

There are some limitations in our study. This is a rather small study of 55 selected pulmonary hypertension patients since 35 were excluded due to atrial fibrillation (*n* = 15) and poor acoustic windows (*n* = 20). However, strain analysis on atrial fibrillation patients are at present not recommended and RV assessment by echocardiography is highly dependent on image quality. Therefore, one could debate which imaging modality is preferred for assessing RV function in this highly selected patient population. Echocardiography and CMR were performed contemporaneously, but not immediately so. Since RV function parameters are load-dependent, small changes in treatment (e.g. diuretic) may affect RV function parameters. However, median time was only 1 day with no cardiac events, medical changes, or clinical deterioration between examinations. FWS_echo_ was measured using an algorithm developed for the left ventricle. However, this method is standard procedure and has been propagated into guidelines [3, 44]. On the other hand, FWS_CMR_ was assessed in RV dedicated algorithm. Further standardization between different vendors for both CMR and echocardiography are required [34] as well as further studies before their utilization in routine clinical practice.

## Conclusion

This study has demonstrated a moderate to strong correlation of regional CMR measurements to corresponding echocardiographic measures. However, biases and to some extent wide limits of agreement, exist between the modalities. Consequently, the equivalent measures are not interchangeable at least in patients with pulmonary hypertension. The echocardiographic parameter that showed best correlation with RVEF_CMR_ was FWS_echo_. At present, FAC_echo_ and FWS_echo_ as well as EF_CMR_ are the preferred methods to assess and follow up RV function in patients with pulmonary hypertension. Future investigations of the CMR right ventricular measures, beyond RVEF, are warranted.

## Supplementary information

**Additional file 1: Table S1.** Differences in characteristics and ventricular function parameters between gender, comorbidities and aetiology of pulmonary hypertension.

## Data Availability

The datasets generated and/or analysed during this study are not available for publications due to research subject confidentiality. They are available in a unidentified form from the corresponding author on reasonable request.

## Abbreviations

CMR: Cardiac magnetic resonance
Echo: Echocardiography
EF: Ejection fraction
RV: Right ventricle
FAC: Fractional area change
FWS: Free wall strain
AVPD_lat_: Lateral atrio-ventricular plane displacement
S’_CMR_: Maximum emptying velocity
S’_echo_: S’-wave velocity
TAPSE: Tricuspid annular plane systolic excursion
