# Supplementary material for: Right ventricular function parameters in pulmonary hypertension: echocardiography vs. cardiac magnetic resonance
Source: BMC Cardiovasc Disord. 2020 Jun 1;20:259. doi: 10.1186/s12872-020-01548-4 (PMC7268227; doi:10.1186/s12872-020-01548-4)
Supplement: Supplementary file 1 — Additional file 1: Table S1. Differences in characteristics and ventricular function parameters between gender, comorbidities and aetiology of pulmonary hypertension. [file 12872_2020_1548_MOESM1_ESM.docx]

**Supplemental Table 1**. Differences in characteristics and ventricular function parameters between gender, comorbidities and aetiology of pulmonary hypertension.

|  | **Gender** | | | **Comorbidities** | | | **PAH** |  |  |
| --- | --- | --- | --- | --- | --- | --- | --- | --- | --- |
| **Parameters** | **Women (n=38)** | **Men**  **(n=17)** | **p** | **Yes**  **(n=26)** | **No**  **(n=29)** | **p** | **Yes**  **(n=40)** | **No**  **(n=15)** | **p** |
| IVSd (mm) | 9.9±2.3 | 9.6±2.7 | n.s | 9.5±2.5 | 10.1±2.3 | n.s | 10±2.5 | 9.3±2.2 | n.s |
| LVPWd (mm) | 8.6±2.2 | 9.1±1.7 | n.s | 8.5±2.3 | 9.0±1.7 | n.s | 8.8±2.0 | 8.7±2.0 | n.s |
| LVEDV (ml) | 65±30.1 | 99±35 | p=0.001 | 77±23 | 76±34 | n.s | 75±33 | 81±44 | n.s |
| LVESV (ml) | 25±17 | 44±21 | p=0.003 | 33±22 | 30±19 | n.s | 29±18 | 38±25 | n.s |
| LVEF (%) | 62±13 | 56±12 | n.s | 58±14 | 62±11 | n.s | 62±13 | 56±12 | n.s |
| LA volume/BSA (ml/m^2^) | 28±15 | 27±13 | n.s | 31±15 | 25±12 | n.s | 27±14 | 30±14 | n.s |
| RA volume/BSA (ml/m^2^) | 41±26 | 34±14 | n.s | 45±26 | 33±18 | n.s | 37±19 | 44±31 | n.s |
| RA area (cm^2^) | 21±8 | 24±5 | n.s | 24±7 | 20±7 | n.s | 21±6 | 24±9 | n.s |
| RV size inflow (mm) | 47±9 | 50±8 | n.s | 49±8 | 46±10 | n.s | 48±9 | 47±7 | n.s |
| RV size mid cavity (mm) | 37±9 | 41±12 | n.s | 40±9 | 35±11 | n.s | 38±11 | 38±7 | n.s |
| TR gradient (mmHg) | 56±21 | 55±23 | n.s | 58±21 | 53±21 | n.s | 55±23 | 57±17 | n.s |
| **RV function parameters** |  |  |  |  |  |  |  |  |  |
| RVEF (%) | 42±14 | 41±13 | n.s | 40±14 | 43±14 | n.s | 41±14 | 42±11 | n.s |
| AVPD_lat_ (mm) | 13±4 | 14±4 | n.s | 13±4 | 14±5 | n.s | 14±4 | 13±4 | n.s |
| S´_CMR_ (cm/s) | 8.6±2.3 | 9.4±3.4 | n.s | 8.7±3.6 | 9.0±2.4 | n.s | 8.6±2.6 | 9.4±4 | n.s |
| FAC_CMR_ (%) | 36±15 | 33±10 | n.s | 34±13 | 37±14 | n.s | 35±13 | 36±14 | n.s |
| FWS_CMR_ (%) | -20.3±7.9 | -19.6±7.4 | n.s | -17.9±7.6 | -22.5±7.1 | p=0.025 | -20.0±7.5 | -20.2±8.3 | n.s |
| TAPSE (mm) | 19±6 | 19±6 | n.s | 19±6 | 19±6 | n.s | 19±6 | 19±6 | n.s |
| S´_echo_ (cm/s) | 11.1±3.5 | 11.6±3.6 | n.s | 11.0±3.8 | 19.5±6.0 | n.s | 11.1±3.4 | 11.7±3.9 | n.s |
| FAC_echo_ (%) | 32±14 | 27±12 | n.s | 27±13 | 33±14 | n.s | 30±14 | 31±14 | n.s |
| FWS_echo_ (%) | -16.1±5.0 | -14.8±4.4 | n.s | -15.0±4.4 | -16.5±5.1 | n.s | -15.1±4.7 | -17.4±4.9 | n.s |

Data is expressed as mean±SD. PAH (pulmonary arterial hypertension), IVSd (intra ventricular septum diameter, LVPWd (left ventricular posterior wall diameter), LVEDV (left ventricular end-diastolic volume), LVEF (left ventricular ejection fraction), LVESV (left ventricular end-systolic volume), LA (left atrium), RA (right atrium), RV (right ventricle), TR gradient (trans-tricuspid gradient), RVEF (right ventricular ejection fraction derived from CMR), AVPD_lat_ (lateral atrio-ventricular plane displacement), S´ (S´-wave velocity), FAC (fractional area change), FWS (right ventricular free wall strain), TAPSE (tricuspid annular plane systolic excursion).
